# Feasibility of using cross-sectional area of masticatory muscles to predict sarcopenia in healthy aging subjects

**DOI:** 10.1038/s41598-024-51589-4

**Published:** 2024-01-24

**Authors:** Aleksa Janović, Biljana Miličić, Svetlana Antić, Đurđa Bracanović, Biljana Marković-Vasiljković

**Affiliations:** 1 School of Dental Medicine, Center for Diagnostic Imaging, University of Belgrade, 6 Rankeova, 11000 Belgrade, Republic of Serbia; 2 School of Dental Medicine, Department of Statistics, University of Belgrade, 2 dr Subotića, 11000 Belgrade, Republic of Serbia

**Keywords:** Medical research, Diseases

## Abstract

Determination of sarcopenia is crucial in identifying patients at high risk of adverse health outcomes. Recent studies reported a significant decline in masticatory muscle (MM) function in patients with sarcopenia. This study aimed to analyze the cross-sectional area (CSA) of MMs on computed tomography (CT) images and to explore their potential to predict sarcopenia. The study included 149 adult subjects retrospectively (59 males, 90 females, mean age 57.4 ± 14.8 years) who underwent head and neck CT examination for diagnostic purposes. Sarcopenia was diagnosed on CT by measuring CSA of neck muscles at the C3 vertebral level and estimating skeletal muscle index. CSA of MMs (temporal, masseter, medial pterygoid, and lateral pterygoid) were measured bilaterally on reference CT slices. Sarcopenia was diagnosed in 67 (45%) patients. Univariate logistic regression analysis demonstrated a significant association between CSA of all MMs and sarcopenia. In the multivariate logistic regression model, only masseter CSA, lateral pterygoid CSA, age, and gender were marked as predictors of sarcopenia. These parameters were combined in a regression equation, which showed excellent sensitivity and specificity in predicting sarcopenia. The masseter and lateral pterygoid CSA can be used to predict sarcopenia in healthy aging subjects with a high accuracy.

## Introduction

Sarcopenia is defined as age-related progressive and generalized loss of skeletal muscle mass (SMM) and strength^1^. The clinical and scientific interest in sarcopenia has dramatically increased in recent years due to the growing proportion of elderly individuals and the high personal, social, and economic burden of this condition^2^. Sarcopenia is associated with many adverse health outcomes in older adults, such as frailty, physical disability, multisystemic organ disease, poor quality of life, and mortality^2^. Loss of SMM and sarcopenia that occur secondary to malignancy have been confirmed as an independent poor prognostic factor for treatment complication risk and survival^3,4^.

Diagnostic imaging has become the most popular and widely used method to diagnose sarcopenia in research and clinical settings. For this purpose, computed tomography (CT) imaging has been accepted as the gold standard^5^. Sarcopenia is typically defined on CT indirectly by quantifying skeletal muscle cross-sectional area (CSA) on a single slice at the level of the third lumbar vertebra (L3)^6^. Obtained CSA divided by stature (height in m^2^) determines skeletal muscle index (SMI), which indicates the whole-body SMM. In cases when CT images at the L3 level are not diagnostically available, there are suggested alternative vertebral levels that correlate well with SMI at L3^5^. For the head and neck region, CSA of sternocleidomastoid and paravertebral muscles at the level of the third cervical vertebra (C3) has been extensively used for sarcopenia estimation due to its strong association with CSA at the L3 level^7,8^.

There is much evidence to support the use of masticatory muscles in sarcopenia analysis. Recent studies reported a significant decline in masticatory function associated with sarcopenia in elderly subjects^9–11^. Some studies detected the link between masticatory muscle quantity and adverse health outcomes in various clinical conditions. The temporal muscle thickness measured on magnetic resonance imaging (MRI) was successfully used as a prognostic marker in patients with glioblastoma, brain metastases, and various neurological disorders^12–16^. Some authors found an association between low masseter mass and mortality in elderly patients after trauma^17–19^. In contrast, others did not confirm the prognostic relevance of the masseter for the same clinical condition^20,21^. CT-assessed low masseter mass has also been linked to postoperative pneumonia in oesophageal cancer^22^, carotid endarterectomy^23,24^, and stroke outcome^25^. However, the feasibility of using medial and lateral pterygoid muscles for the same purpose has not been investigated.

This study explored whether masticatory muscles, single or in combination, may be suggested for sarcopenia estimation. We tested hypotheses that: (1) CT-assessed CSA of masticatory muscles differs significantly between subjects with and without sarcopenia, (2) masticatory muscles CSA correlates significantly with SMI, and (3) masticatory muscles CSA may be used as a predictor of sarcopenia. All masticatory muscles were included in the analysis (temporal, masseter, medial pterygoid, and lateral pterygoid) in order to explore their individual potential in sarcopenia analysis.

## Methods

### Study design and participants

This cross-sectional study was conducted retrospectively at the Center of Diagnostic Imaging, University of Belgrade, School of Dental Medicine. The approval for study conduction was obtained from the Ethical Committee of the same institution, decision No. 36/44. The study was performed in accordance with the ethical standards of the responsible committee on human experimentation and the Declaration of Helsinki. The informed consent was obtained from all study participants.

**Figure 1:**
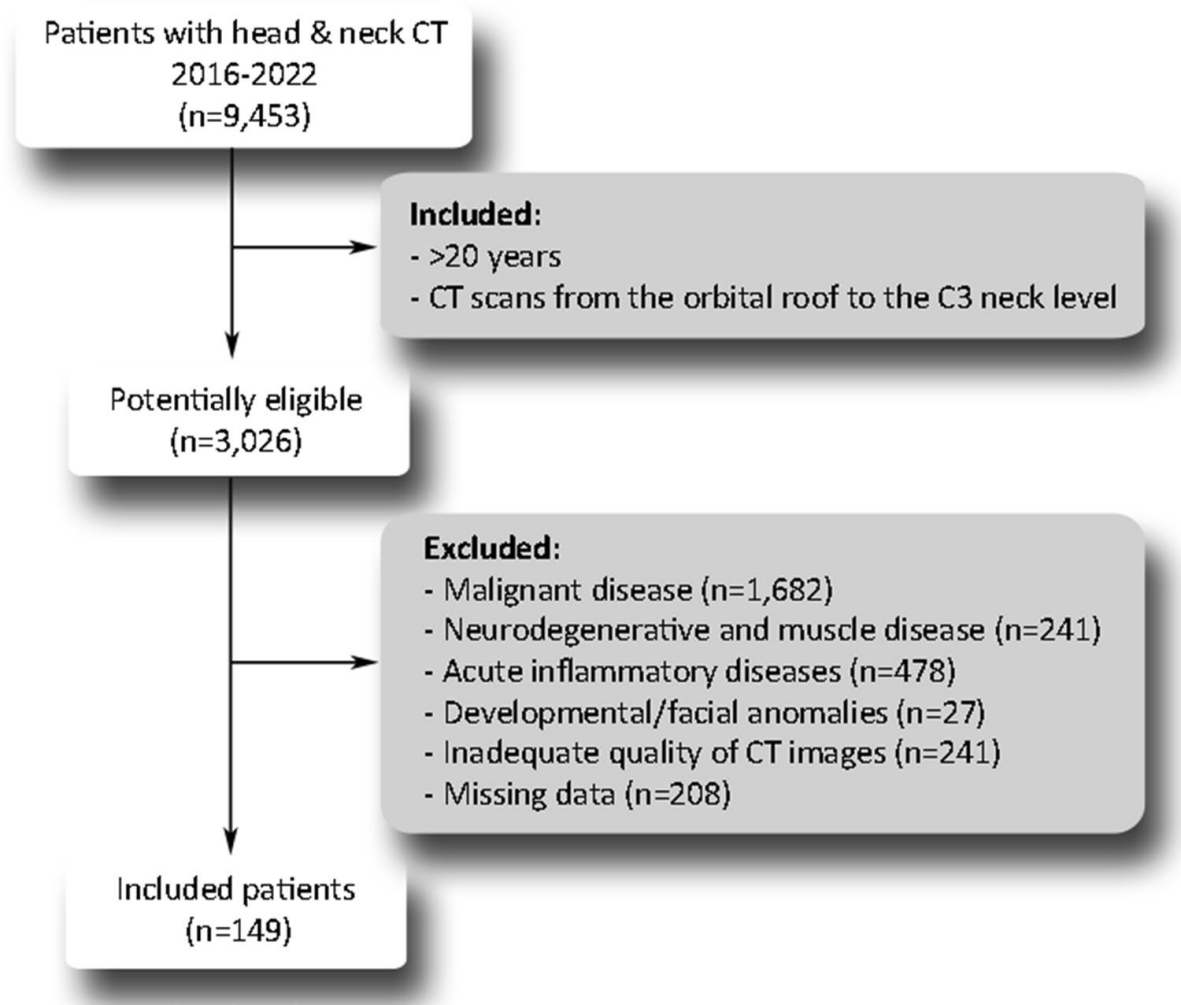
The flow chart of patient selection.

Study participants were selected among patients who underwent head and neck CT examinations for diagnostic purposes between January 2016 and December 2022. All patients were referred to CT examination by a maxillofacial surgeon, oral surgeon, or ear-nose-throat specialist due to various head and neck diseases. CT images were stored at the Picture Archive and Communications System (PACS). Patient selection criteria are shown in Fig. 1. Patients potentially eligible for the study were selected based on the following inclusion criteria: (1) patients > 20 years, and (2) CT examination contains scans from the orbital roof to the C3 level of the neck. During the evaluation of medical records and CT images, patients were excluded if one of the following conditions was found: (1) a history of malignant disease, (2) neurodegenerative and muscle disease, (3) developmental facial anomalies, (4) extensive inflammation of the neck soft tissues that obscure muscle borders, and (5) missing data. Scans with insufficient quality and severe metal artifacts from tooth restorations were also excluded from the analysis. The final sample consisted of 149 patients in whom various benign conditions of the head and neck region were diagnosed. The most common conditions were benign salivary gland tumors, various benign tumors of the head and neck soft tissues (e.g. lipoma), developmental cysts of the head and neck soft tissues, jaw bone cysts, and odontogenic tumors. Since these conditions cannot cause or accelerate sarcopenia development, study participants were therefore considered “healthy”.

All CT examinations were performed using a Philips Ingenuity Core 64-raw CT device (Philips Medical Systems, Cleveland, USA). Patients were positioned with the head resting on the holder to ensure a stable and uniform position of the head and neck. Scanning was performed with a slice thickness of 1 mm, tube current of 50–120 mA, and voltage of 90–140 kV. Tube current and voltage were adjusted to the patient's body weight.

Age (in years), gender, body height (in m), body weight (in kg), and body mass index (BMI, in kg/m^2^) were recorded. A number of missing posterior teeth was also counted during the evaluation of CT images.

### CT diagnosis of sarcopenia

A routine protocol for SMI estimation was used to diagnose sarcopenia on head and neck CT scans^7,8,26^. A single axial slice was selected at the C3 level with the first entire vertebral arch, including spinous and transverse processes visible when scrolling in the caudo-cephalad direction^7^. CSA (in cm^2^) of paravertebral and sternocleidomastoid muscles were quantified bilaterally in Osirix software (v9.0; Pixmeo SARL, Bernex, Switzerland). Muscles were selected by applying a standard Hounsfield unit (HU) threshold from – 29 to + 150 and manual delineation. The CSA at the C3 level was measured by a board-certified radiologist with extensive experience in head and neck imaging (S.A.) supervised by a senior radiologist (B.M.V.).

Obtained CSA at C3 was used to estimate CSA at L3 using the following equation^7^:

$${\text{CSA at L3 }}\left( {{\text{cm}}^{{2}} } \right) \, = { 27}.{3}0{4 } + { 1}.{363}*{\text{CSA at C3 }}\left( {{\text{cm}}^{{2}} } \right) \, - \, 0.{671}*{\text{age }}\left( {{\text{years}}} \right) \, + \, 0.{64}0*{\text{weight }}\left( {{\text{kg}}} \right) \, + { 26}.{442}*{\text{sex }}\left( {{\text{Sex }} = {\text{ 1 for female}},{\text{ 2 for male}}} \right).$$

CSA at L3 was normalized by height in m^2^ to calculate SMI (cm^2^/m^2^). The following sex- and BMI-specific cut-off values for SMI were applied to classify patients as sarcopenic or not: < 53 cm^2^/m^2^ for overweight or obese males, < 43 cm^2^/m^2^ for underweight or healthy weight males, and < 41 cm^2^/m^2^ for females^27^.

### CT analysis of masticatory muscles

**Figure 2:**
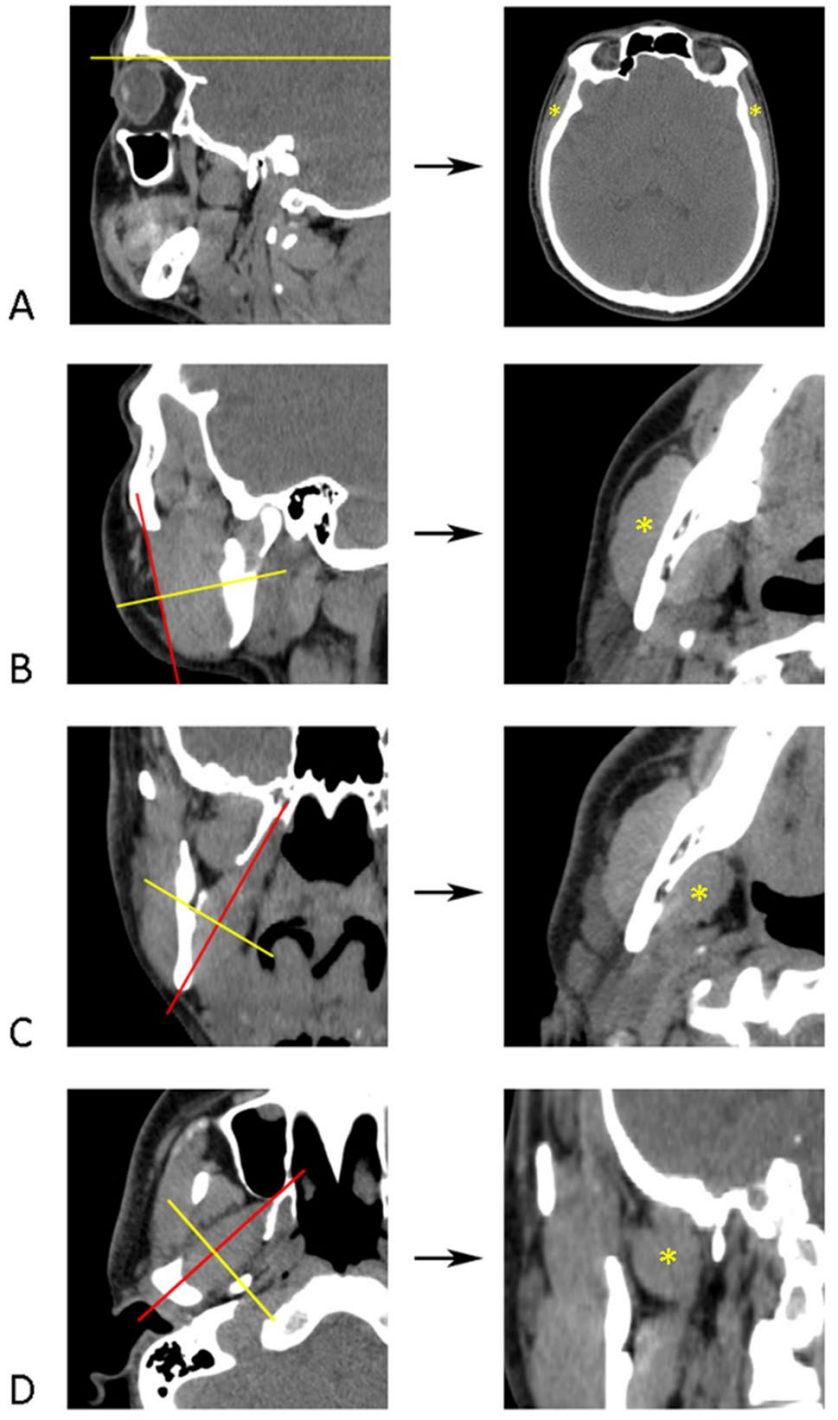
Selection of reference CT images of masticatory muscles for CSA measurement. (**A**) Axial CT image at the level of the superior orbital margin (yellow line) used as a reference image for the temporal muscle measurement. (**B**) The reference CT slice at the level of the mandibular foramen (yellow line) for the masseter measurement was selected perpendicular to the muscle long axis (red line). (**C**) For the medial pterygoid, the selected CT image at the level of the mandibular foramen (yellow line) was perpendicular to the muscle’s long axis (red line connects the inferior margin of the mandible and the top of the pterygoid fossa). (**D**) The reference CT slice for the lateral pterygoid measurement was selected at the middle of the muscle (yellow line) perpendicular to the muscle’s long axis (red line).

CSA (in cm^2^) of temporal, masseter, medial pterygoid, and lateral pterygoid muscle was measured bilaterally. A single CT slice at the specific level was selected for each muscle in relation to the bone anatomical landmarks. Axial CT slices were first aligned with the zygomatic arch and scrolled from the caudal to the cephalad direction. The superior orbital margin was selected as a reference level for the temporal muscle CSA measurement (Fig. 2A). This level was chosen in order to include muscle tissue without tendon. Axial CT slices were then scrolled caudally to the level of the masseter. The axial plane was aligned with the masseter anterior margin in order to fit its long axis (Fig. 2B). The first oblique axial CT slice showing the mandibular foramen when scrolling in the caudal-cephalad direction was selected for CSA measurement (Fig. 2B). The medial pterygoid CSA was measured on the right and left sides separately. On the right side, the muscle-long axis was estimated on the frontal CT slice showing the mandibular foramen (Fig. 2C). The plane connecting the inferior border of the mandibular ramus and the top of the pterygoid fossa (the lateral and medial pterygoid process fusion) corresponded to the muscle-long axis. An oblique axial CT slice perpendicular to the muscle's long axis at the level of the mandibular foramen was selected for CSA measurement (Fig. 2C). The same procedure was repeated for the left medial pterygoid. The long axis of the lateral pterygoid was estimated on the axial CT slice by connecting the center of the mandibular condyle and the pterygomaxillary fissure (Fig. 2D). An oblique frontal CT section at the midpoint of the muscle, perpendicular to the long axis, was selected for the CSA measurement.

The masticatory muscle measurements were performed independently by two other board-certified radiologists (A.J. and D.B.), who were blinded to the SMI measurement outcome. The same software and HU threshold values were used. The sum of CSAs of the right and the left side was calculated for each muscle and assigned as total CSA (tCSA). The mean tCSA values from the two radiologists were used for analysis.

### Statistical analysis

Collected data were analyzed in SPSS software (version 25.0, Inc., Chicago, IL). Parametric variables (age, tCSA of masticatory muscles) were presented as mean and standard deviation. The normality of the data distribution was assessed by the Kolmogorov–Smirnov test. Frequencies were calculated for nonparametric data (gender, presence of sarcopenia). Differences in masticatory muscle tCSA between groups were explored using Unpaired Student’s *t* test. Pearson correlation analysis was performed to explore the relationship between tCSA of masticatory muscles and SMI. Univariate and multivariate logistic regression models evaluated the relationship between age, gender, and masticatory muscles as dependent variables and sarcopenia as an independent variable. Odds ratio (OR) and 95% confidence interval (CI) were calculated. Receiver operating characteristic (ROC) curve analysis was performed. The prognostic performances of selected parameters were evaluated according to the area under the curve (AUC). The level of significance was set to 0.5.

## Results

One hundred and forty-nine patients were included in the study (59 (39.6%) males, 90 (60.4%) females). Demographic and anthropometric features of patients are summarized in Table 1. The mean age ± standard deviation of mean was 57.42 ± 14.78 years, ranging between 25 and 86. Sarcopenia was diagnosed in 67 (45%) patients, predominantly in females (Table 1). The mean age of patients with and without sarcopenia was 62.55 ± 10.99 and 53.23 ± 16.15 years, respectively (Table 1). Concerning BMI, most patients were in the overweight category. Patients with sarcopenia had significantly higher BMI. The groups also differed significantly in the mean number of missing teeth (Table 1). The same was confirmed for CT-assessed SMI, which was significantly lower in the sarcopenia group.

**Table 1:** Demographic, anthropometric, and CT image data with comparative analysis between groups.

Parameter	Total sample (n = 149)	Sarcopenia		*p* value
		Present (n = 67)	Absent (n = 82)	
Age	57.42 ± 14.78	62.55 ± 10.99	53.23 ± 16.15	< 0.001*
Gender				
Male	59 (39.6%)	7 (10.4%)	52 (63.4%)	< 0.001**
Female	90 (60.4%)	60 (89.6%)	30 (36.6%)	
BMI (kg/m^2^)	26.83 ± 4.96	24.35 ± 3.37	28.86 ± 5.15	< 0.001*
BMI category				
< 20.0	6 (4.0%)	5 (7.5%)	1 (1.2%)	
20.0–24.9	52 (34.9%)	32 (47.8%)	20 (24.4%)	
25.0–29.9	62 (41.6%)	28 (41.8%)	34 (41.5%)	
≥ 30.0	29 (19.5%)	2 (2.9%)	27 (32.9%)	
Missing teeth				
Mean	8.96 ± 5.70	10.72 ± 5.19	7.52 ± 5.74	0.001*
Median	9	12	7	
Min–max	0–16	0–16	0–16	
CT image analysis				
SMI	43.64 ± 8.87	35.98 ± 4.30	49.89 ± 6.38	< 0.001*
tCSA_TM_	7.38 ± 2.82	3.06 ± 0.98	4.03 ± 1.11	< 0.001*
tCSA_MM_	7.16 ± 2.24	3.04 ± 0.81	4.12 ± 1.15	< 0.001*
tCSA_MPM_	5.52 ± 1.27	2.53 ± 0.49	2.89 ± 0.61	< 0.001*
tCSA_LPM_	6.40 ± 1.49	2.94 ± 0.49	3.54 ± 0.84	< 0.001*

Table 1 also displays the mean values of masticatory muscles tCSA in two patient groups. Patients with sarcopenia had significantly lower tCSA of all masticatory muscles when compared to patients without sarcopenia. A Pearson correlation analysis demonstrated a strong association between tCSA of all masticatory muscles and SMI (Table 2).

**Table 2:** Pearson correlation analysis between masticatory muscles and estimated skeletal muscle index.

Parameter	SMI
tCSA_TM_	0.687*
tCSA_MM_	0.689*
tCSA_MPM_	0.500*
tCSA_LPM_	0.576*

*SMI* skeletal muscle index, *tCSA*_*TM*_ total cross-sectional area of the temporal muscle, *tCSA*_*MM*_ total cross-sectional area of the masseter, *tCSA*_*MPM*_ total cross-sectional area of the medial pterygoid, *tCSA*_*LPM*_ total cross-sectional area of the lateral pterygoid.

*Correlation is significant at the 0.01 level (2-tailed).

*BMI* body mass index, *SMI* skeletal muscle index, *tCSA*_*TM*_ total cross-sectional area of the temporal muscle, *tCSA*_*MM*_ total cross-sectional area of the masseter, *tCSA*_*MPM*_ total cross-sectional area of the medial pterygoid, *tCSA*_*LPM*_ total cross-sectional area of the lateral pterygoid.

*Significant difference confirmed by Student’s *t* test. **Significant difference confirmed by Chi-square test.

tCSA of all masticatory muscles showed a significant association with sarcopenia in the univariate logistic regression analysis (Table 3). In the multivariate logistic regression model, however, only masseter and lateral pterygoid tCSA were significantly associated with sarcopenia (Table 3). Age and gender were significantly associated with sarcopenia in univariate and multivariate logistic regression models, whereas number of missing teeth was associated with sarcopenia only in the univariate regression model (Table 3). tCSA of masseter and lateral pterygoid remained significant predictors when adjusting for variance explained by age and gender.

**Table 3:** Univariate and multivariate logistic regression models for assessed parameters and sarcopenia.

Parameter	Univariate model		Multivariate model	
	OR (95% CI)	*p*	OR (95% CI)	*p*
Age	1.050 (1.023–1.078)	** < 0.001**	1.128 (1.062–1.199)	** < 0.001**
Gender	14.859 (6.025–36.639)	** < 0.001**	7.672 (1.869–31.494)	**0.005**
tCSA_TM_	0.529 (0.423–0.661)	** < 0.001**	1.149 (0.782–1.681)	0.484
tCSA_MM_	0.436 (0.326–0.585)	** < 0.001**	0.480 (0.288–0.800)	**0.005**
tCSA_MPM_	0.421 (0.296–0.600)	** < 0.001**	0.884 (0.502–1.558)	0.670
tCSA_LPM_	0.477 (0.352–0.647)	** < 0.001**	0.536 (0.327–0.878)	**0.013**
Missing teeth	1.238 (1.137–1.349)	** < 0.001**	1.136 (0.818–1.578)	0.446

*OR* odds ratio, *CI* confidence interval, *tCSA*_*TM*_ total cross-sectional area of the temporal muscle, *tCSA*_*MM*_ total cross-sectional area of the masseter, *tCSA*_*MPM*_ total cross-sectional area of the medial pterygoid, *tCSA*_*LPM*_ total cross-sectional area of the lateral pterygoid.

Significant values are in bold.

**Figure 3:**
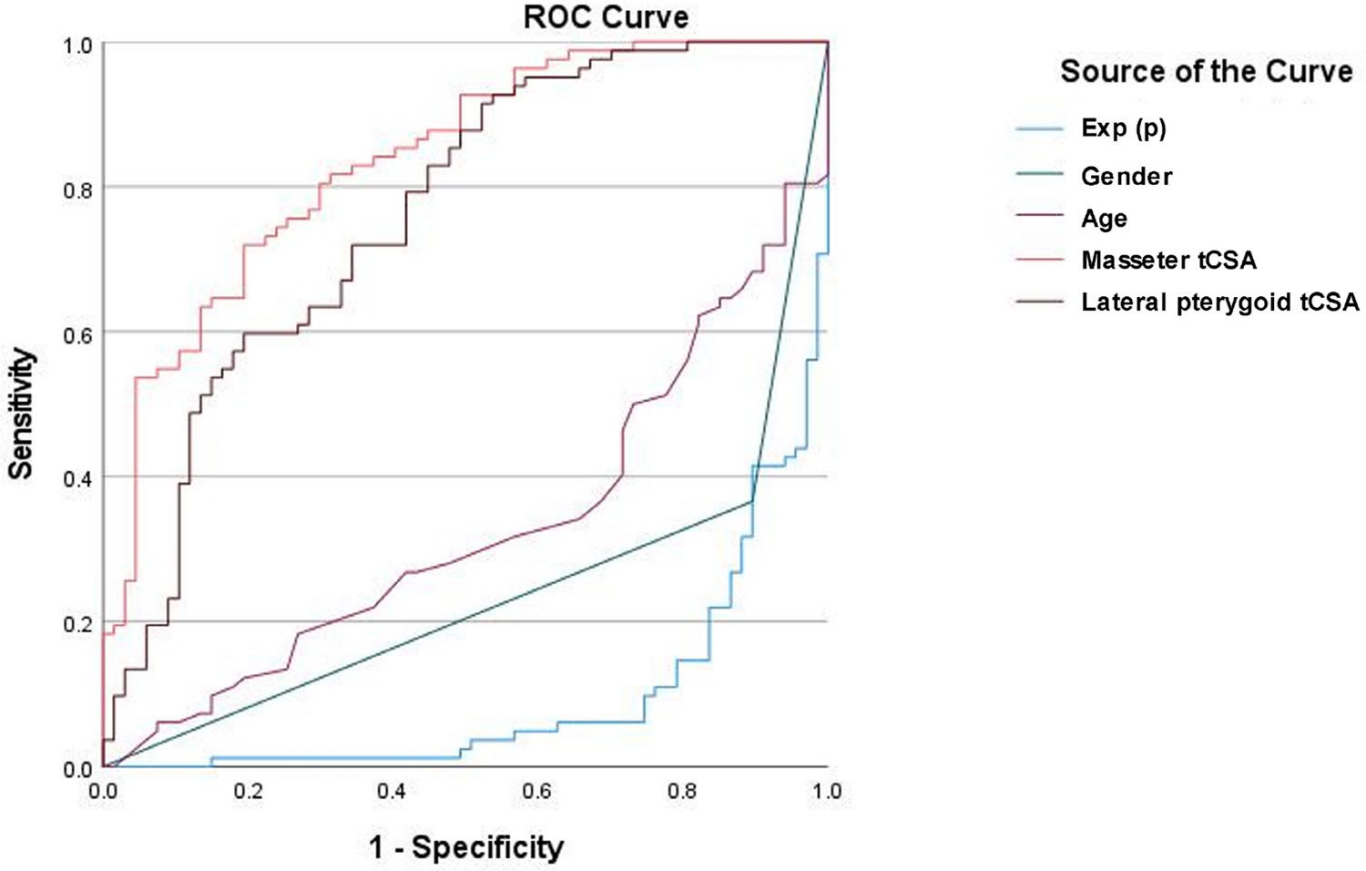
Receiver operating characteristics curve of sarcopenia using age, gender, tCSA of the masseter muscle, and tCSA of the lateral pterygoid muscle. The sensitivity and specificity of the calculated predictive model (Exp(p)) are also illustrated.

The results of the ROC curve analysis are summarized in Table 4 and illustrated in Fig. 3. Masseter tCSA showed the highest diagnostic accuracy (AUC = 0.837) among the analyzed parameters. It was the best parameter for correctly classifying patients with and without sarcopenia. Good diagnostic accuracy was obtained for tCSA of the lateral pterygoid muscle (AUC = 0.761) and gender (AUC = 0.765). Age showed satisfactory diagnostic accuracy (AUC = 0.670). Results of the multivariate logistic regression analysis were used to define a predictive model of sarcopenia. The following equation was made:

$${\text{Exp }}\left( {\text{p}} \right) \, = \, - {2}.0{67 } + { 2}0{38}*{\text{gender }}\left( {{\text{1 for male}},{\text{ 2 for female}}} \right) \, + \, 0.{121}*{\text{age }}{-} \, 0.{733}*{\text{tCSA}}_{{{\text{MM}}}} {-} \, 0.{624}*{\text{tCSA}}_{{{\text{LPM}}}} .$$

**Table 4:** Receiver operating characteristics analysis of sarcopenia prediction using age, gender, CSA of the masseter muscle, and the CSA of the lateral pterygoid muscle.

Parameter	AUC	p	95% CI
Age	0.670	< 0.001	0.584–0.756
Gender	0.765	< 0.001	0.687–0.843
tCSA_MM_	0.837	< 0.001	0.774–0.900
tCSA_LPM_	0.761	< 0.001	0.683–0.839
Exp (p)	0.906	< 0.001	0.856–0.954

*AUC* area under the curve, *CI* confidence interval, *tCSA*_*MM*_ total cross-sectional area of the masseter, *tCSA*_*LPM*_ total cross-sectional area of the lateral pterygoid, *Exp(p)* probability of the sarcopenia presence.

The probability of the sarcopenia presence (p) can be calculated by the equation: p = Exp (p)/(1–Exp(p)). The equation result may have different value ranging from 0 to 1 (0–100%). For example, if the equation result is 0.9 (or 90%), it suggests the high probability of sarcopenia presence, and vice versa. The ROC curve analysis showed excellent diagnostic accuracy for the predictive model (Table 4, Fig. 3), which can correctly classify up to 95.4% of patients into a group with or without sarcopenia.

## Discussion

Determination of SMM and sarcopenia is of tremendous importance in identifying patients at high risk of potential adverse health outcomes. Besides the well-established method of SMM assessment on CT images at the L3 level, many studies tested other vertebral levels that may serve as an accurate alternative in sarcopenia analysis when abdominal CT is not routinely performed. The use of alternative vertebral levels was almost exclusively investigated in patients with cancer-related sarcopenia. The current study analyzed the CSA of all masticatory muscles in healthy aging subjects in order to explore their individual potential in predicting sarcopenia. We decided to analyze CSA because this parameter is strongly associated with masticatory muscle strength and determines the maximum bite force that each muscle can produce^28–30^.

Our results revealed significant morphological changes in all masticatory muscles associated with sarcopenia. As displayed in Table 1, tCSA of all masticatory muscles differed significantly between patients with and without sarcopenia. This finding is expected when considering a thigh connection between mastication and nutrition. Masticatory muscles are the main determinants of occlusal forces and chewing ability that are, with other oral functions, necessary to maintain adequate food intake. Impaired masticatory function due to any reason may change the food selection by avoiding the food that is hard to chew, e.g. meat, fruit, and vegetables^31,32^. Thus, elderly subjects with impaired mastication are at higher risk of developing malnutrition and consequent sarcopenia due to low uptake of proteins and other essential nutrients^10^.

Although correlation analysis indicates that a significant age-related decrease in the quantity of all masticatory muscles coincides with the decrease of SMI, masticatory muscles do not have the same potential to predict sarcopenia. Multivariate logistic regression analysis marked masseter and lateral pterygoid muscle as significant predictors of sarcopenia. Among all muscles, masseter tCSA was the best parameter in distinguishing patients with and without sarcopenia, with very good sensitivity and specificity (Table 4). The masseter muscle is a strong rectangular muscle whose origin lies on the zygomatic arch, and insertion is on the mandibular angle^33^. During mastication, this muscle pulls the mandible upward, thus providing contact between upper and lower teeth and generating occlusal forces. Changes in masseter muscle quantity have been recently linked to body composition parameters, which are frequently affected in patients with sarcopenia. Masseter CSA was positively associated with BMI, hand grip strength, and walking speed^34,35^, whereas it was negatively associated with body fat content^36^. Masseter CSA was also marked as a valuable parameter in predicting early and late mortality in older patients after blunt injury^17,19,37,38^. Van Heusden et al. found that masseter CSA is significantly associated with overall survival in patients with head and neck cancer^39^. Only Chang et al.^38^ tested the feasibility of using masseter CSA in estimating SMI at the L3 level in the Asian population with head and neck cancer patients. The authors introduced a masseter-SMI by dividing CSA with squared height and correlated this parameter with L3-SMI^38^. The measurement of CSA was performed at the level of the mandibular notch. Chang et al. concluded that masseter-SMI can be used as an alternative in detecting sarcopenia in head and neck cancer^38^.

However, the masseter muscle is not the only masticatory muscle that can be used to predict sarcopenia in the healthy aging population. The lateral pterygoid muscle has been neglected in the sarcopenia analysis, although its quantity has recently been reported to be positively associated with BMI and hand grip strength^36^. As demonstrated by Daboul et al.^40^, the CSA of this muscle gradually decreases during aging, especially in women. Our study demonstrated a significant predictive potential of the lateral pterygoid muscle for sarcopenia. To our knowledge, this study is the first to highlight the importance of using tCSA of the lateral pterygoid muscle in sarcopenia analysis. The lateral pterygoid muscle is a jaw-opening muscle located in the infratemporal fossa between the mandible and the pterygoid process of the sphenoid bone^33^. Its bilateral contraction protrudes the mandible, while the unilateral contraction is responsible for lateral movement and rotation of the mandible. It contributes to mastication by stabilizing the temporomandibular joint during biting and chewing. The lateral pterygoid muscle is always scanned during routine CT imaging of the head and neck and maxillofacial region. Moreover, its appearance on CT is not affected by scattering artifacts from dental restorations and metal implants.

The changes in medial pterygoid muscle morphology also occur during aging. Newton^41^ detected a gradual decrease in CSA of the medial pterygoid muscle in aging subjects. Daboul et al.^40^ recently confirmed that the CSA of the medial pterygoid is significantly dependent on age, but the effect of gender was uncertain. In the current study, the morphology of the medial pterygoid was analyzed in the context of sarcopenia for the first time. Our results suggest that subjects with generalized sarcopenia have a significant depletion in CSA of the medial pterygoid, which positively correlated with SMI. However, changes in medial pterygoid CSA did not show enough sensitivity and specificity to be used in the sarcopenia prediction. The same can be concluded for the temporal muscle. Although the temporal tCSA in our study was reduced significantly in patients with sarcopenia, changes in this parameter were poorly associated with the diagnosis of sarcopenia. Many recent studies confirmed the use of temporal muscle thickness as a surrogate marker of sarcopenia due to its strong association with treatment outcomes and mortality in oncology patients^12–16^. By contrast, our study does not support the use of temporal CSA in sarcopenia analysis in healthy aging subjects.

Other analyzed parameters, such as age and gender, showed significant predictive potential in sarcopenia diagnosis but with different specificity and sensitivity. Gender showed good diagnostic accuracy, similar to CSA of the lateral pterygoid. Various factors, such as genetic and hormonal, determine the differences in the whole-body muscle mass in males and females. In general, females have a lower muscle mass than males. The cut-off values for sarcopenia calculation based on SMI are also lower in females^27^.

Interestingly, age showed the lowest specificity and sensitivity for sarcopenia prediction among analyzed parameters. One of the reasons could be non-uniform tooth loss across ages. It has been shown that masticatory function and masticatory muscle properties are closely connected with the number of teeth^34,42–45^. The tooth loss decreases the strength of the masticatory muscles, especially the masseter, and may cause muscle thinning^34,43,44^. Such functional and structural deterioration in masticatory muscles may further lead to malnutrition and consequently increase the risk for sarcopenia. The goal of the current study was not to correlate tooth loss with masticatory muscle properties. As has already been demonstrated in many studies, every change in teeth number will cause more or less deterioration in masticatory muscle morphology^34,42–45^. Therefore, assessment of masticatory muscles CSA at any time during aging will be in accordance with the current dental status. Additionally, a stronger association between age and sarcopenia was not obtained because tooth loss may be treated with various tooth resorations or dental implants during life and thus prevent or prolongue the onset of malnutrition and sarcopenia.

The results of our study may have direct diagnostic and therapeutic implications. Masseter and lateral pterygoid CSA may be suggested as an accurate alternative to CSA at the C3 level in estimating sarcopenia in patients referred to CT examination of the head and neck. The equation obtained from the multivariate logistic regression showed an excellent predictive potential ranging up to 95%, which makes it valid to use in patients with head and neck CT. Early detection of patients at risk of developing sarcopenia would provide timely application of preventive measures to minimize masticatory muscle depletion, improve mastication, and ensure adequate nutrition.

The current study highlighted the potential of using masticatory muscles for skeletal muscle mass prediction and sarcopenia, but some potential limitations need to be addressed. Although this method could be easily applied to both CT and MR studies, limitations may arise on CT images in patients with extensive tooth restorations and/or metal implants. Scattering artifacts from metal objects may obscure borders of the masseter muscle in the region of interest and thus introduce a bias in the CSA measurement. However, this could be overcome by changing the head position during scanning and/or by applying software that may significantly reduce metal artifacts from the CT image. A potential bias may arise from the study sample, which is relatively small and heterogeneous, particularly when considering the gender distribution of sarcopenia. Therefore, our results might not be representative of a large population. The study was performed on healthy aging subjects and might not have the same potential in predicting skeletal muscle mass and sarcopenia in patients with head and neck cancer. Sarcopenia in malignant diseases might follow different mechanisms. Further studies are needed to investigate the individual effect of tumor-related cachexia on different muscle groups, such as masticatory muscles and paravertebral muscles. Additionally, since there is no consensus among researchers on which sarcopenia cut-off values should be used, we considered sex- and BMI-specific cut-off values proposed by Marin et al.^23^ as the most sensitive and most appropriate for our population.

## Conclusion

All masticatory muscles showed significant differences in CSA between subjects with and without sarcopenia. A significant positive association between masticatory muscles CSA and skeletal muscle index suggests simultaneous deterioration of both muscle groups during aging. Our study revealed a significant predictive value of masseter, lateral pterygoid muscle, gender, and age for sarcopenia. The regression equation generated in this study showed excellent sensitivity and specificity in predicting sarcopenia. This time, we demonstrated that it is possible to diagnose sarcopenia in healthy aging subjects with a high accuracy using CSA of the masseter and lateral pterygoid muscle. Our future studies will investigate whether the same accuracy can be achieved by applying this method to sarcopenia diagnosis in oncology patients.

## Data Availability

All data generated or analysed during this study are included in this published article.
